# The use of CytoSorb hemoadsorption in critically ill patients: a narrative review

**DOI:** 10.3389/fmed.2025.1628241

**Published:** 2025-09-22

**Authors:** Kaixin Lei, Ao Chen, Xinqi An, Jun Guo, Baihai Su, Yupei Li

**Affiliations:** ^1^Department of Nephrology, Kidney Research Institute, West China Hospital of Sichuan University, Chengdu, China; ^2^Department of Critical Care Medicine, West China Hospital, Sichuan University, Chengdu, China; ^3^Med+ Biomaterial Institute of West China Hospital/West China School of Medicine, Sichuan University, Chengdu, China; ^4^Med-X Center for Materials, Sichuan University, Chengdu, China

**Keywords:** hemoperfusion, CytoSorb®, critical illness, inflammation, mortality

## Abstract

Inflammation, a complex biological response against injury or infection, is an important pathological basis for various critical diseases. A “normal” immune response exemplifies a balanced dialogue between immunological cells and a medley of pro- and anti-inflammatory mediators. However, under pathological conditions, this equilibrium is disrupted by the overwhelming release of cytokines, also known as a cytokine storm, which significantly contributes to multiple organ dysfunction and death. Accumulating clinical evidence highlights the efficacy of CytoSorb® hemoadsorption in eliminating damage-associated molecular patterns, pathogen-associated molecular patterns and excessive cytokines from the blood, which underscores the broad use of CytoSorb® in managing various critical conditions. In this narrative review, we conduct a state-of-the-art review of CytoSorb® hemoadsorption in daily critical care practice. By searching ‘CytoSorb®’, ‘cytokine absorption’, ‘hemoadsorption’ and ‘hemoperfusion’ in PubMed, Embase and Web of Science, we discussed the rationale and research progress for cytokine adsorption with CytoSorb® from January 2019 to May 2025. Then, we summarize the latest clinical evidence regarding the use of CytoSorb® in sepsis, cardiac surgery, extracorporeal membrane oxygenation, hepatic diseases, rhabdomyolysis and burn injuries. Finally, we elaborated on the impact of CytoSorb® on the clearance rates of antibiotics and anticoagulants to address its safety concerns and highlighted ongoing debates on the timing, dose and patient selection criteria of CytoSorb® hemoadsorption, which requires future research to optimize actual benefits.

## 1 Introduction

Critical illnesses, including sepsis, severe COVID-19, infective endocarditis, operation-related complications, and burn injuries, are significantly associated with immune dysfunction and subsequent multiple organ dysfunction, such as acute kidney injury (AKI), acute respiratory distress syndrome (ARDS), heart failure, and liver failure (1, 2). The mortality rate of critically ill patients is estimated to reach 15–30% or even higher without proper treatment (3). Accumulating evidence has shown that the progression of life-threatening critical illnesses is associated with the dysregulation of cytokines (1, 4). For example, an overwhelming release of proinflammatory cytokines such as interleukin-1 (IL-1), IL-6, and tumor necrosis factor-*α* (TNF-α) can trigger hyperinflammation and cause ARDS and death in patients with severe COVID-19 (4).

In addition to traditional antibiotic therapy and fluid resuscitation therapy, the elimination of excessive cytokines is a feasible therapeutic strategy to manage hyperinflammation-related critical diseases in intensive care units (ICUs) (5). Over the past two decades, hemoperfusion, a common extracorporeal blood purification technique, has been applied in ICUs worldwide to remove pro-inflammatory mediators from the bloodstream (6). Hemoperfusion uses a mechanism of adsorption to eliminate both proinflammatory and anti-inflammatory cytokines to modulate the dysregulated host immune response (7). Recently, evolutionary techniques have led to the development of more biocompatible and potentially more efficient adsorptive materials for daily critical care practice (6, 7).

**Figure 1:**
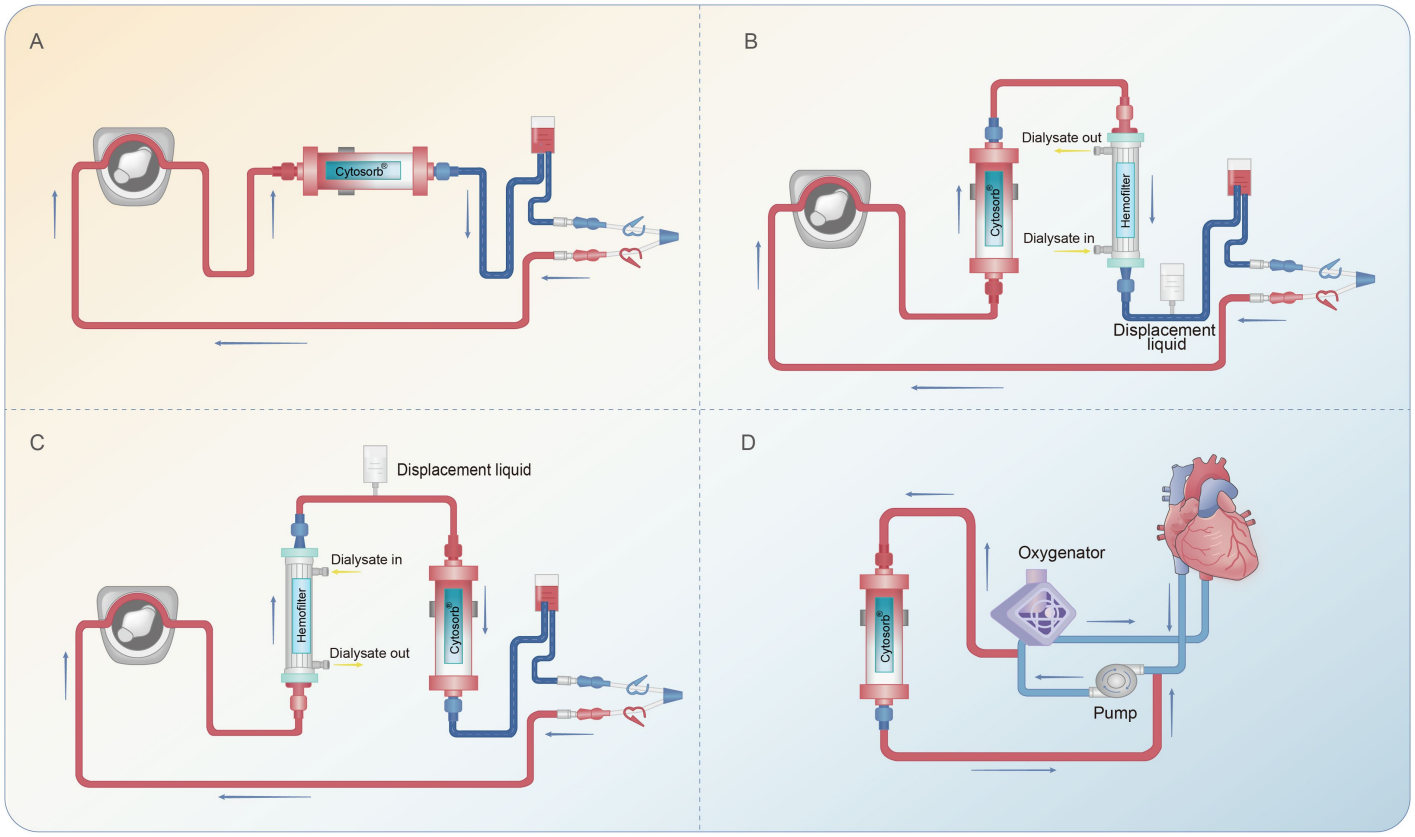
A schematic diagram of CytoSorb® in an extracorporeal blood purification circuit. **(A)** CytoSorb® used alone for hemoadsorption. **(B)** CytoSorb® attached as pre-dialyzer. **(C)** CytoSorb® attached as post-dialyzer. **(D)** CytoSorb® incorporated with an extracorporeal membrane oxygenation circuit or cardiopulmonary bypass.

Among them, CytoSorb® (CytoSorbents Corporation, New Jersey, USA), a hemoperfusion cartridge engineered to eliminate deleterious enterotoxin, cytokines, bilirubin and myoglobin (5, 8, 9), has been marketed in 53 countries across the globe and been indicated for a wide range of hyperinflammation-associated severe diseases (8, 10). Primarily composed of polystyrene-divinylbenzene copolymer beads (5), CytoSorb® employs a combination of size exclusion and hydrophobic interactions to selectively adsorb proteins and cytokines within the molecular weight range of 10 to 60 kDa, including key inflammatory mediators such as TNF-*α*, IL-1 and IL-6, to modulate the hyperinflammatory cascade (11–14). As shown in Figure 1, CytoSorb® can be used alone or in combination with continuous renal replacement therapy (CRRT) or extracorporeal membrane oxygenation (ECMO). The treatment duration can be up to 24 h per day for up to 7 consecutive days, with an optimal blood flow rate ranging from 250 to 400 mL/min. However, CytoSorb® hemoadsorption is contraindicated in patients with a history of heparin-induced thrombocytopenia and unacceptable citrate regional anticoagulation (15).

**Figure 2:**
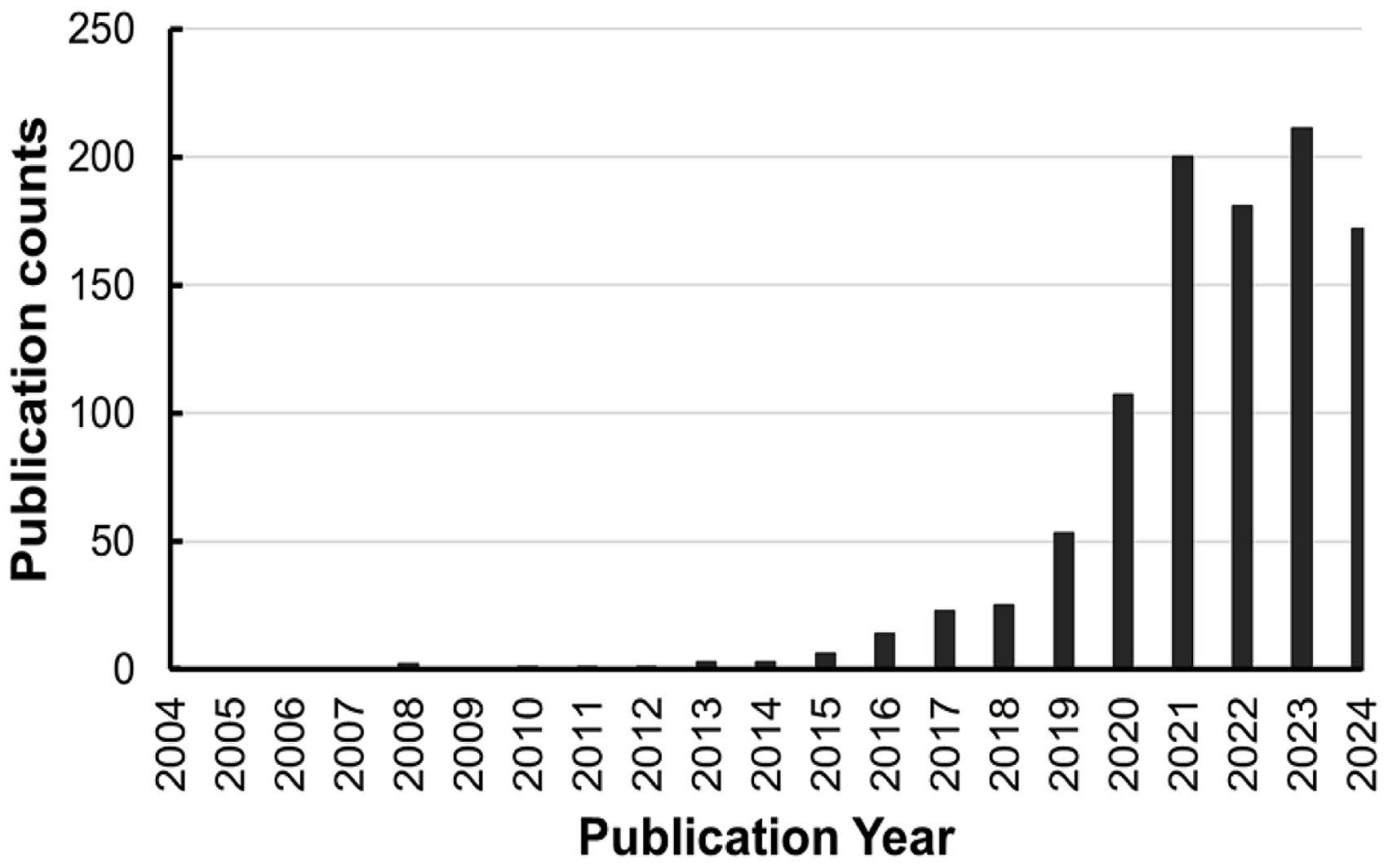
The upcoming focus on CytoSorb® in the past two decade on PubMed.

Several clinical studies have reported favorable outcomes, but a consensus on the influence of CytoSorb® therapy on patient-centered outcomes has yet to emerge. This is largely due to the inherent limitations of currently available trials in this field, including modest sample sizes, considerable variability among participants, and short follow-up durations (16). Furthermore, the capability of CytoSorb® to adsorb a spectrum of toxic substances, such as bilirubin and myoglobulin, has greatly expanded its potential clinical applications recently. However, a comprehensive understanding of the effects of CytoSorb® hemoadsorption on hyperinflammation, hemodynamics, organ function, and mortality in critically ill patients increased dramatically, especially in recent 5 years (Figure 2).

In this narrative review, we extensively summarize the clinical evidence of CytoSorb® treatment in managing sepsis, severe COVID-19, cardiac surgery, liver failure, rhabdomyolysis and burn. Additionally, we elaborate on the impact of CytoSorb® on the clearance rates of antibiotics and anticoagulants to address safety concerns. It is believed that this review will provide a profound understanding of CytoSorb® hemoadsorption in ICU settings.

## 2 Methods

### 2.1 Databases and search strategy

In order to systemically update the latest research progress on cytokine absorption, we searched for articles published from January 2019 to May 2025 in PubMed, Embase, and Web of science following keywords of ‘CytoSorb®’, ‘cytokine adsorption’, ‘hemoadsorption’ and ‘hemoperfusion’. A total of 650 papers were searched as relevant items in recent 5 years. In addition, we also searched clinicaltrials.gov using similar key terms to update ongoing clinical trials, with 10 registries included.

### 2.2 Screening process and article selection

Two authors (Kaixin Lei and Ao Chen) scrutinized the searched articles with language restriction of English. The inclusion criteria are (1) case reports, case series, cross-sectional studies, cohort studies and RCTs reporting cytokine adsorption therapy with CytoSorb® in critically ill patients; (2) experimental *in vitro* or *in vivo* studies elucidating the safety and adsorption kinetics for CytoSorb®; (3) preclinical and clinical studies concerning the effect of CytoSorb® treatment on hard outcomes (e.g., mortality) or surrogate changes (e.g., IL-6 levels) in critically ill patients or animal models. The exclusion criteria were (1) basic studies investigating mechanisms of cytokine elimination; (2) studies without quantitative report or analysis; and (3) cytokine adsorption predominantly achieved by other hemofilters.

**Figure 3:**
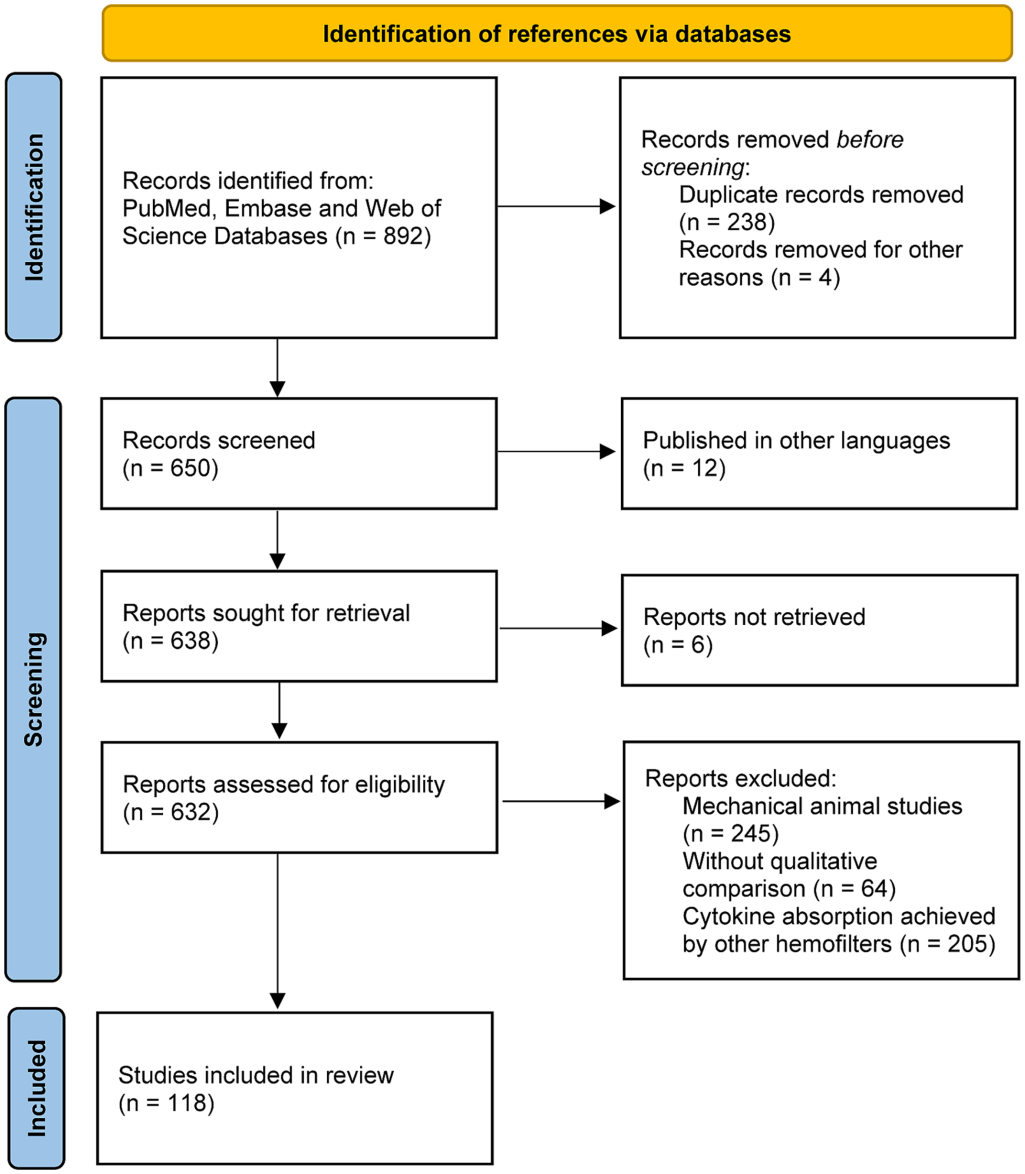
The flowchart of selection procedure of supporting evidence.

Each article was screened by the two authors for agreement and final decision was determined by the corresponding authors if any divergent view was expressed. Ultimately, a total of 118 references were considered as supporting evidence (Figure 3). Furthermore, clinical trials published former than 5 years with large sample sizes and cautious study design were also taken into consideration as these results provided compelling evidence for clinical decision-making procedure.

### 2.3 Quality assessment

For better understanding of qualities from current findings, we employed Risks of Bias 2 (RoB2), Newcastle-Ottawa Scale (NOS) and Joanna Briggs Institute (JBI) Critical Appraisal Checklist to evaluate evidence from RCTs, observational studies and case series, respectively (17, 18).

## 3 Clinical application of CytoSorb® Hemoadsorption in critically ill patients

### 3.1 Sepsis

Sepsis, a leading cause of AKI, is defined as a host dysregulated immune response secondary to infection (19). Pathophysiologically, pattern recognition receptors recognize endotoxins or damage-associated molecular patterns to trigger a dysregulated immune activation of leukocytes, a release of proinflammatory mediators, such as IL-1, IL-6 and TNF-ɑ, into the bloodstream, and finally a cytokine storm (20, 21). High serum concentrations of proinflammatory cytokines are associated with multiple organ dysfunction syndrome (MODS) and mortality (22). Previous preclinical studies reveal that CytoSorb® has a high affinity for IL-6, IL-10, TNF-*α*, monocyte chemoattractant protein-1 (MCP-1) and human neutrophil peptide alpha-defensin 1 (HNP-1) (13, 23–25). Hopefully, CytoSorb® may be helpful for septic patients by eliminating these proinflammatory mediators (19, 26).

Dating back to 2020, Jasen and colleagues recruited 24 healthy male volunteers and randomly assigned them to either the CytoSorb® group or the control group (5). Following the initial administration of endotoxin, there was a significant reduction in the plasma concentrations of TNF-*α* (− 58%, *p* < 0.0001), IL-8 (− 48%, *p* = 0.02), IL-10 (− 26%, *p* = 0.03), and IL-6 (− 71%, *p* = 0.003) in the CytoSorb® group (5). However, this effect was substantially attenuated after the second administration of endotoxin. Additionally, Hawchar and colleagues prospectively included 20 patients with early (< 24 h) septic shock (27). After a 48-h CytoSorb® treatment, patients in the CytoSorb® group demonstrated a significant reduction in norepinephrine requirements (0.16 μg/kg vs. 0.25 μg/kg, *p* = 0.016) and procalcitonin concentrations (5.6 ng/kg vs. 9.2 ng/kg, *p* = 0.004), without adverse events (27). These improvements were most significant during the first 12 h, aligning with discoveries from Jasen (5, 28). These findings suggest that CytoSorb® can reduce short-term systematic inflammation without interfering with long-term immunity.

Other observational studies also reported inconsistent hard outcomes along with a CytoSorb® therapy in patients with sepsis. For example, a recent retrospective study involving 116 individuals demonstrated that septic patients receiving CytoSorb® therapy had a lower risk of 28-day mortality [adjusted HR 0.59, 95% confidential interval (CI) 0.37–0.93, *p* = 0.0025] (29). Similarly, another retrospective cohort study enrolling 70 septic patients in Germany reported a decrease in 28-day mortality (73% vs. 50%, *p* < 0.01) after CytoSorb® hemoadsorption (30). Beyond these, several case studies reported that the average sequential organ failure assessment (SOFA) significantly decreased in CytoSorb® hemoadsorption, delivering potential benefits in maintaining organ functions. However, despite these positive changes of organ functions, the 28-day mortality rates and length of ICU stay were not significantly improved (31–33).

In summary, these evidence on temporary improvements of laboratory variables from observational studies displayed concerns in sample sizes, comparability and confounding factors in different groups, thereby delivering controversial views on long-term patient-centered endpoints such as mortality. Data from case series provided weak evidence despite elaborative description of clinical information of these patients. An updated meta-analysis concluded that CytoSorb® could not improve the long-term survival rate in septic patients, raising concerns about taking CytoSorb® as a routine care (34). Consequently, pertinent suggestions have been consolidated for the consideration of CytoSorb® as a potential adjunctive therapy for septic patients (Table 1) (35–38). As the heterogenous nature of septic patients and insufficient investigations from current data, these suggestions should not be interpreted as universally applicable guideline recommendations. Future studies with randomization, high-quality, larger sample size, multicenter design, and long follow-up duration are needed to substantiate the current findings.

**Table 1:** Pragmatic considerations for CytoSorb® hemoadsorption in patients with vasoplegic shock.

Patients	Septic or septic shock with Cytoscore > 6 (35) (Weak), signs of hyperinflammation, obvious elevation of inflammatory markers, e.g., IL-6 (if detectable) (36, 37) (Moderate)
Timing	Start within 12 h and no longer than 24 h after diagnosis (3) (Moderate)
Dose and frequency of filter change	Initially, change the absorbent medium every 8–12 h during primary 2 days, later renew the medium every 24 h, hemoperfusion until hemodynamic stabilization (38) (Weak)
Accompanying medication	For drugs affine to CytoSorb®, supervise circulating dose or additional dose after initiation of CytoSorb® therapy (10, 143) (Weak). Therapeutic drug monitor is recommended at regular pace

Suggestions that solely based on observation investigations with small sample sizes were identified as weak tier; suggestions based on randomized controlled trials with strict study designs, large prospective cohort study or meta-analysis with clear methodology and conclusions were identified as moderate tier. This table only illustrates clinically practical hypothesis in current literatures, which should be interpreted with caution.

### 3.2 COVID-19

First reported in December 2019, severe acute respiratory syndrome coronavirus 2 (SARS-CoV-2), a member of the beta-coronavirus genus, has caused numerous deaths worldwide. Approximately 20% of COVID-19 patients with ARDS exhibit elevated IL-1β, IL-6, TNF-*α*, C-X-C motif chemokine ligand 10 and chemokine ligand 2 levels, thereby triggering MODS (1, 39, 40). Consequently, cytokine hemoadsorption with CytoSorb® might be useful in patients suffering from severe COVID-19 infection (4, 41, 42).

Since 2021, there have been several pilot reports evaluating the efficacy of CytoSorb® hemoadsorption in critically ill COVID-19 patients and some cases reported successful attempts (43–48). Some retrospective studies demonstrated that CytoSorb® treatment held the potential to improve clinical outcomes of severe COVID-19 patients, albeit to debated outcomes. In regards to mortality, a single-center retrospective study reported that 55 COVID-19 patients undergoing CytoSorb® hemoperfusion had a lower mortality rate than those in the control group (67.3% vs. 89%, *p* = 0.02) (49). Similarly, another study identified CytoSorb® as a feasible strategy for 44 severe COVID-19 patients with AKI compared with 58 patients exclusively receiving CRRT (60-day survival rate, 65.9% vs. 84.5%, *p* = 0.029) (50). In another retrospective study enrolling 26 patients with severe COVID-19, significant reductions in the PaO_2_/FiO_2_ ratio (168.6 vs. 302.0, *p* < 0.01), SOFA score (14.9 vs. 7.4, *p* < 0.01), C-reactive protein (CRP) level (83.1 mg/L vs. 35.5 mg/L, *p* < 0.01) and lactate level (12.1 mg/dL vs. 8.0 mg/dL, *p* < 0.01) were observed following CytoSorb® treatment, with 21 patients survived (45). Additionally, other case studies with small samples showed that CytoSorb® hemoadsorption was effective in improving surrogate outcomes including SOFA score, PaO_2_/FiO_2_ as well as IL-6 levels (46–48). According to NOS and JCI assessments, concerns about selection bias, intergroup comparability, adjustments for confounding factors and insufficient sample sizes or follow-up time weakened reliability of these results.

In contrast, RCTs reported debated results concerning the efficacy of CytoSorb® treatment in severe COVID-19 patients. In 2022, a multicenter randomized controlled trial enrolled 50 patients with COVID-19-induced vasoplegic shock who received either CytoSorb® treatment plus standard medical care for 3 to 7 days (CytoSorb® changed for every 24 h) or standard medical care. There were no statistically significant differences in mortality rate (78% vs. 73%, *p* = 0.64), IL-6 level on day 3 (66.3 ng/L vs. 103.0 ng/L, *p* = 0.78) between the CytoSorb® group and the control group (51). Likewise, another single-center RCT involving 24 patients with COVID-19-induced vasoplegic shock also reported no significant differences in SOFA score (17 vs. 16, *p* = 0.55), median IL-6 concentration (2,269 ng/L vs. 3,747 ng/L, *p* = 0.38), or 28-day mortality rate (58% vs. 67%, *p* = 1.0) after CytoSorb® therapy (CytoSorb® was indicated continuously for 5 days with replace every 24–48 h) (52). In summary, the small sample size of existing studies, heterogeneity of therapy prescriptions or patient selection, and different follow-up periods are key factors contributing to the debate over the efficacy of CytoSorb® in critically ill COVID-19 patients (53). Therefore, large-scale RCTs are necessitated to explore the effect of CytoSorb® on patient-centered outcomes, including mortality rates and SOFA scores, and underlying positive prognostic factors.

### 3.3 Cardiac surgery

Cardiac surgeries, including heart transplantation, valve surgeries and aortic surgeries, increase the risk systemic inflammatory response syndrome owing to hypoperfusion, ischemia–reperfusion injury, neuro-humoral activation and intraoperative or postoperative infections (54, 55). *In vitro*, CytoSorb® efficiently eliminated inflammatory mediators and regulated vascular endothelial damage (56, 57), thus warranting attention for peri-operation management.

Since 2018, several clinical studies have investigated the efficacy of combined CytoSorb® therapy in patients undergoing cardiac surgery. For instance, a proof-of-concept RCT that included 55 patients undergoing orthotopic heart transplantation showed that patients in the CytoSorb® hemoadsorption group had lower vasoactive-inotropic scores (41.9 vs. 27.2, *p* = 0.046) and vasoplegic syndrome incidence rates (48% vs. 20%, *p* = 0.022) than those in the control group (58). Additionally, in patients undergoing orthotopic heart transplantation, CytoSorb® treatment was associated with lower need for norepinephrine and blood transfusion (59, 60). Meanwhile, Kristina et al. reported that, in 98 patients with AKI and septic shock after cardiac surgery, CytoSorb® hemoadsorption contributed to a decrease in the vasoactive score (56.7 vs. 26.7, *p* < 0.001) and in-hospital mortality (77% vs. 53%, *p* < 0.001) (61). However, 30-day mortality and 1-year survival rates did not exhibit any differences. Owing to relatively high risks based on NOS, these findings partially suggested short-term hemodynamic improvements in cardiac surgery patients.

The prognosis of CytoSorb® hemoadsorption for cardiac surgery remain controversial. Studies involving 130 endocarditis patients undergoing surgical interventions (CytoSorb® *n* = 75, Control *n* = 55) demonstrated that patients in the CytoSorb® group had lower vasoactive-inotropic score (6 vs. 17, *p* = 0.0014), sepsis related mortality (8.0% vs. 22.8%, *p* = 0.02), 30-day mortality (17.3% vs. 32.7%, *p* = 0.03) and 90-day mortality (21.3% vs. 40%, *p* = 0.03) (62, 63), while a preliminary RCT that included 30 patients undergoing elective cardiac surgeries observed no substantial changes in the levels of pro- or anti-inflammatory cytokines after peri-operative CytoSorb® hemoadsorption (9). Moreover, the CytoSorb® intervention exhibited insignificant changes in clinical hard endpoints. The multicenter REMOVE trial recruited 288 infective endocarditis patients in 14 cardiac centers in German and assigned them into the CytoSorb® hemoadsorption (*n* = 142) and control (*n* = 146) groups during cardiac surgery (64). After a 30-day follow-up, there were no differences in SOFA changes (1.79 ± 3.75 vs. 1.93 ± 3.53, *p* = 0.6766) and 30-day mortality rates (21% vs. 22%, *p* = 0.782) between the CytoSorb® group and the control group, despite IL-1β and IL-18 levels decreased at 30 min, 60 min and the endpoint after initiation of hemoadsorption (64). Even in intention-to-treat analysis, these outcomes were not significant as well, indicating a limited therapeutic prospect in combating infective endocarditis. However, the therapeutic plan of REMOVE trial was not standardized. Compared with other studies indicated CytoSorb® as adjunctive treatment for more than 3 days in peri-operative managements, REMOVE trial only used CytoSorb® alone during cardiac surgery for several hours, which may shadow the authentic efficacy of this device.

Overall, current evidence suggests that the use of CytoSorb® therapy during and after cardiac surgery may temporarily improves hemodynamics. Notwithstanding the noted decreases in inflammatory cytokines like IL-1 and TNF-*α*, patient-centered outcomes, such as SOFA and 30-day mortality, do not appear to be strikingly manifest. All observational studies displayed moderate to high risks in regard to heterogeneity of participants and hemoadsorption prescription. This divergence between biochemical improvements and clinical benefits underscores the need for further investigations into the precise pathways linking cytokine reduction to long-term clinical outcomes of patients (9, 58, 64–66).

### 3.4 Extracorporeal membrane oxygenation

During the last decade, extracorporeal membrane oxygenation (ECMO) is indicated for patients with lethal clinical syndromes such as vasoplegia and MODS (67–69). Nonetheless, a body of research indicates that ECMO procedures can induce inflammatory responses due to the activation of mast cells after interaction between artificial surfaces and blood components (70, 71). As a consequence, the use of CytoSorb® in combination with ECMO is proposed (67–70).

In 2023, a retrospective study consecutively included 359 patients with refractory cardiac arrest under mechanical chest compression (*n* = 120), profound cardiogenic shock (*n* = 101), post-cardiotomy cardiogenic shock (*n* = 81), respiratory failure (*n* = 34) and COVID-19 infection (*n* = 15) to evaluate the effect of combined ECMO and CytoSorb® hemoadsorption on mortality rates. The results showed that the mortality rates at 30 months, within the ICU, and during the hospital stay were 49, 57, and 62%, respectively, which were lower than the mortality predictions of 71% as estimated by the simplified acute physiology score II and the 68% as forecasted by the SOFA score (72). In other small sample case series, CytoSorb® treatment during ECMO was reported to significantly improve inflammatory parameters (73–76). Besides, in cardiogenic shock patients, the combination of CytoSorb® and V-A-ECMO therapy reduced vasopressor requirements, lactate levels, and mortality, with increased urine output and decreased need for CRRT (77, 78).

However, some RCTs and prospective cohort studies conflicted with these positive findings from retrospective studies. In 2020, the CYCOV trial, the first single open-label RCT, divided 34 COVID-19 patients requiring ECMO into two groups (79). After 72 h of CytoSorb® hemoadsorption, the median IL-6 concentration decreased from 357.0 pg/mL to 98.6 pg/mL in the CytoSorb® group and from 289.0 pg/mL to 112.0 pg/mL in the control group, but the difference was not significant (*p* = 0.54). Notably, 14 patients died in the CytoSorb® group, compared to only 3 in the control group, suggesting a potential negative effect of CytoSorb® treatment on survival (79). This may be traced to the immunosuppression after improper clearance of cytokines, potentially exacerbating the damage caused by infection. Additionally, inappropriate on-broad time and incomparable baseline IL-6 level (357.0 pg/mL in CytoSorb® group vs. 289.0 pg/mL in the control group) may account for higher death burden in CytoSorb® group. A subsequent single-center trial including 50 patients who received ECMO for extracorporeal cardiopulmonary resuscitation reported no improvements in serum IL-6, survival, vasopressor support, or markers of injury (80). In further post-hoc analysis in 41 patients of this trial, the alternation of IL-6 was still not significant. Moreover, a study enrolling 21 patients with out-of-hospital cardiac arrest demonstrated that in 10 patients receiving hemoadsorption proved that CytoSorb® therapy failed to reduce IL-6 levels without safety concerns (81).

In summary, the prognosis of CytoSorb® in conjunction with ECMO remains insufficiently explored. The observational studies so far have very small sample sizes, raising the possibility of small sample bias, while compelling RCTs with standard CytoSorb® prescription (e.g., the on-broad time and frequency of changing hemofilters) and comparable baseline characteristics are still absent, arousing concerns for current conclusions. ECMO is used in the ICU for a variety of conditions, and the effects of combining ECMO with CytoSorb® in different clinical scenarios are not yet well understood. Patients most suitable for this technique and prognostic factors that may play a role in selecting optimal population (e.g., SOFA) are controversial. Although CytoSorb® exhibited some pessimistic results in prognosis, future research could cultivate potential beneficial subgroups with longer follow-up periods, larger sample sizes and standardized designs.

### 3.5 Liver failure

Liver failure can cause metabolic imbalances favoring hepatic bilirubin production over enteric or uric clearance (82, 83). Excessive bilirubin, a dominant target of CytoSorb® hemoadsorption, in the serum of liver failure patients is toxic and can further cause extensive destruction of the liver, kidney, heart and skin (82, 84, 85). Riva et al. demonstrated that CytoSorb® had the superior adsorption capability for bilirubin and bile acids in comparison with the molecular adsorbent recirculating system, the fractionated plasma separation and adsorption system (86). *In vitro* studies also showed that CytoSorb® exhibited an outstanding ability to adsorb bilirubin (87, 88).

Following several successful case series (89–91), observational studies investigated prospective outcomes of CytoSorb® in patients with liver failure. Greimel et al. prospectively included 20 ICU patients with cholestatic liver disorders and integrated CytoSorb® into the dialysis circuit, measuring total and conjugated bilirubin levels (92). Initially, the reduction ratios for total and conjugated bilirubin were −31.8% and −30.3% and these ratios decreased to −4.5% and −4.8% respectively, after 6 h (92). Another study including 33 acute liver failure patients reported a median reduction ratio of total bilirubin of 22.8% after 1 day of CytoSorb® therapy (93). Similarly, Haselwaner et al. retrospectively analyzed 21 patients with acute-on-chronic liver failure and found that, after CytoSorb® hemoadsorption, serum levels of bilirubin, procalcitonin and IL-6 decreased significantly from 20.7 mg/L to 10.8 mg/L (*p* < 0.001), from 1.34 pg/L to 0.74 pg/L (*p* < 0.001), and from 385 ng/L to 131 ng/L (*p* = 0.0182), respectively (94). In contrast, a prospective, randomized, single-center, open-label, controlled pilot trial (CYTOHEP) aimed to investigate the effect of bilirubin absorption by CytoSorb® hemoadsorption (95). Patients with acute-on-chronic liver failure were divided into three groups: CRRT with hemoadsorption, CRRT alone, and no CRRT. After 72 h of extracorporeal hemoperfusion, the median level of bilirubin in the combined CRRT and CytoSorb® group was lowered by −8.0 mg/dL (*p* = 0.17) compared with that in the CRRT alone group. When comparing CRRT with hemoadsorption to no CRRT, the reduction was not significant (−9.4 mg/dL, 95% CI, −20.8 to 2.1 mg/dL; *p* = 0.0854). These results failed to prove the efficacy of CytoSorb® in eliminating bilirubin for patients with acute-on-chronic liver failure. Nevertheless, due to difficulties in recruiting patients and ethical concerns, the CYTOHEP trial was terminated early with only 9 patients, and the open-label study design may also influence the results. Consequently, this study exhibited high risks and insufficiently provided robust evidence, which demanded further exploration in RCTs and cohorts with larger sample sizes.

As for mortality, Gräfe et al. reported that CytoSorb® hemoadsorption was not associated with improved survival rates in 82 patients with bilirubin levels greater than 10 mg/dL (96). RCTs with mortality as primary outcome measure are thus needed in the future to justify the use of CytoSorb® in patients with hyperbilirubinemia.

### 3.6 Rhabdomyolysis

Rhabdomyolysis refers to straited muscle damage or necrosis that results in the leakage of intracellular components into the extracellular fluid (97). During rhabdomyolysis, destroyed muscle cells can release myoglobin and creatine kinase, which further disrupt renal tubular integrity and ultimately trigger AKI through the Fenton reaction (98, 99). Early in 2015, Wiegele et al. reported the first use of CytoSorb® hemoadsorption in a patient with legionella pneumonia–associated rhabdomyolysis. They found that CytoSorb® treatment significantly reduced plasma myoglobin from 18,390 to 10,020 ng/mL within 8 h to preserve renal function (100). Since then, CytoSorb® hemoadsorption has been implemented as a therapeutic alternative for rhabdomyolysis in several case reports (101–103).

Scharf et al. subsequently performed a retrospective study enrolling 43 critically ill rhabdomyolysis patients with myoglobin levels higher than 5,000 ng/mL who underwent CytoSorb® hemoadsorption for more than 90 min (104). They reported that there was a significant correlation between creatine kinase and myoglobin at all measurement points. In 21 patients without ongoing rhabdomyolysis, the median circulating myoglobulin concentration significantly decreased by 38% during CytoSorb® treatment. Additionally, Albrecht et al. included 8 participants and randomly assigned them into two equal groups (105). The area under the curve for myoglobin concentration was significantly reduced at 24 h (42 ± 10% vs. 63 ± 6%, *p* = 0.029) and 48 h (26 ± 7% vs. 51 ± 12%, *p* = 0.029) in patients treated with CytoSorb® (105). In contrast, CytoSorb® hemoadsorption failed to reduce myoglobin levels in 22 patients with increased creatine kinase and ongoing rhabdomyolysis, with a median relative reduction of only 4%.

In 2024, Graf et al. conducted a prospective study that included 20 severe rhabdomyolysis patients with plasma myoglobin levels higher than 5,000 ng/mL to further determine the adsorption capacity and saturation kinetics of myoglobin elimination (106). The median myoglobin plasma clearance at 10 min after CytoSorb® treatment was 64.0 ml/min, decreasing rapidly to 29.1 mL/min, 16.1 ml/min, 7.9 mL/min, and 3.7 mL/min after 1, 3, 6, and 12 h, respectively. In the following year, a prospective cohort consist of 102 patients with rhabdomyolysis and AKI who underwent treatment with CytoSorb in combination with high-flux F60S dialyzer demonstrated significant improvement of SOFA score despite elimination of myoglobulin (107). Similarly, Caroline et al. investigated 35 matched pairs of patients with a myoglobin concentration >10,000 ng/mL (108). After the 30-day follow-up, the kidney recovery rate was significantly higher in the CytoSorb® group compared to the control group (61.1% vs. 23.5%, *p* = 0.03). In general, some cohorts considered potential effects from confounding factors and kept intergroup comparability to offer convincing observational results (106–108). However, the relatively small sample size, and selection bias from retrospective study design were inevitable. Future research ought to call on controlled prospective designs to sustain these findings.

### 3.7 Burn injuries

Sepsis and septic shock are common complications of severe burns and are associated with high mortality. Patients with severe burns may also develop AKI due to inflammation and microcirculatory dysregulation secondary to sepsis (109). It is well established that burn patients experience an uncontrolled, dysregulated host response characterized by significant changes in mediators such as IL-8, MCP-1, and IL-6, as well as the activation of the apoptosis pathway (110). Consequently, extracorporeal blood purification techniques have been used to treat septic shock in burn patients, addressing both AKI and hyperinflammation (110).

In 2017, the RESCUE trial enrolled 37 burn patients with septic shock and AKI to evaluate the impact of high-volume hemofiltration (HVHF) on hemodynamics and organ function (111). The study found a reduction in vasopressor dependency after 48 h of HVHF treatment at a dose of 70 mL/kg/h and a decrease in the MODS score at 14 days. However, there were no significant differences in survival or changes in inflammatory markers between the HVHF and control groups.

Recently, Mariano et al. conducted a retrospective analysis of the impact of the adjunctive CytoSorb® cartridge in burn patients with septic shock-associated AKI undergoing CRRT (112). The study included 37 burn patients who developed septic shock-associated AKI and received CRRT for more than 72 h. Among them, 11 patients were treated with CytoSorb® as adjunctive therapy for refractory septic shock (Hemoadsorption group), while 24 patients were not (Control group). In the hemoadsorption group, CytoSorb® and CRRT were coupled, with the CytoSorb® cartridge placed in a prefilter position according to the manufacturer’s instructions. The CytoSorb® cartridge and extracorporeal circuit were changed every 24 h. The results showed patients in the hemoadsorption group had a significant reduction in norepinephrine use compared with those in the control group. The in-hospital mortality rates were 45.4 and 70.8% in the hemoadsorption group and control group, respectively. However, these findings, which are not conclusive, should be viewed as a starting point for future randomized controlled trials aimed at clarifying the role of CytoSorb® in the treatment of severe burn patients.

### 3.8 Detoxication

Due to its chemical affinity, CytoSorb® is also used for managing drug overdoses and detoxification. Drugs with similar hydrophobic structures can bind tightly to the polymer beads in the CytoSorb® cartridge (113, 114). For example, lethal doses of digoxin and clozapine can be effectively cleared (115–117). CytoSorb® hemoadsorption is primarily an adjunctive treatment for emergencies and should not replace direct antidote (113, 118–125) (Table 2). Given the differences in drug properties, treatment duration, and blood flow, these parameters should be individually adjusted for personalized treatment (2, 8, 126, 127).

**Table 2:** Drugs that can be absorbed by CytoSorb® and possible effects after clearance.

Possible effect	Drug classification	Pharmacal substance	Reference
Positive or negative effects (depending on indications and dose)	Positive inotropic drugs	Digitoxin	(116)
		Digoxin	(117)
		Levosimendan	(127)
	Anticoagulants	Dabigatran	(128)
		Endoxaban	(129)
		Apixaban	(127, 135)
		Ticagrelor	(130)
		Argatroban	(127, 131)
		Rivaroxaban	(132)
		Bivalirudin	(142)
Positive effects (mainly detoxication)	Antipsychotic drugs	Quetiapine	(119)
		Clozapine	(115)
	Anti-epileptic drugs	Lamotrigine	(120, 127)
		Carbamazepine	(117, 127)
		Phenytonin	
	Antidepressants	Venlafaxine	(121)
		Amitriptyline	(122)
		Amitryptilin	(123)
	Narcotics	3,4-Methylenedioxy-methamphetamine (MDMA)	(124)
		Patent Blue V (diethylamino-4-phenyl)	(125)
	Toxins	Aflatoxin B1	(114)
		Toxic Shock Syndrome toxin-1 (TSST-1)	(13)
Negative effects (disturb therapeutic pharmacokinetics)	Antibacterial drugs	Levofloxacine	(139)
		Ceftazidime	
		Vancomycin	(140)
		Meropenem	(118)
		Ciprofloxacin	
		Clindamycin	(136)
		Linezolid	(137)
	Antimycotic drugs	Fluconazole	
		Posaconazole	
	Antiviral drugs	Remdesivir	(138)
	Plasma components	Albumin	(141)
		Platelet	(141)

The data are mainly collected from clinical trials and case series, and few come from in vitro experiments.

## 4 Drug clearance during CytoSorb® sessions

### 4.1 Anticoagulant removal

Previous studies have reported anticoagulants, including rivaroxaban, edoxaban, apixaban, ticagrelor, and dabigatran etexilate, can be removed by CytoSorb® treatment (128–132). Therefore, CytoSorb® is indicated for alleviating circulating anticoagulant levels and risks for bleeding (Table 2). In patients undergoing cardiac surgery, those treated with CytoSorb® experienced a significant reduction in postoperative bleeding events, platelet transfusion requirements, and postoperative chest tube drainage volume compared to those receiving standard medical care (133–135). These findings suggest that CytoSorb® can improve outcomes for patients undergoing cardiac surgery. However, whether actively removing anticoagulants can reduce serious perioperative bleeding in patients undergoing urgent cardiac surgery requires further evaluation in double-blind randomized studies.

### 4.2 Elimination of anti-infective drugs

The influence of CytoSorb® on the metabolism of anti-infective drugs was reported in 2019. In a case involving a 14-year-old boy with a methicillin-resistant Staphylococcus infection, the dose of clindamycin used during CytoSorb® hemoadsorption had to be adjusted (136). Since then, *in vitro* experiments have demonstrated that CytoSorb® can effectively remove anti-infective drugs (118, 137–141) (Table 2). As for clinical evidence, Scandroglio et al. investigated the impact of CytoSorb® on the kinetics of vancomycin and bivalirudin in 89 patients who underwent CytoSorb® treatment with no significant removal of vancomycin or bivalirudin during CytoSorb® sessions (142). In contrast, a prospective observational study including 7 patients and 160 serum samples suggested that infusing 500 mg of vancomycin over 2 h of CytoSorb® treatment is necessary to avoid subtherapeutic concentrations due to the accelerated drug elimination (10). Overall, CytoSorb® hemoadsorption may accelerate unwanted drug elimination in critically ill patients. However, the degree of clearance is heterogeneous among different drugs, making further studies with emphasis on specific drug targets essential (143).

## 5 Outlook

**Figure 4:**
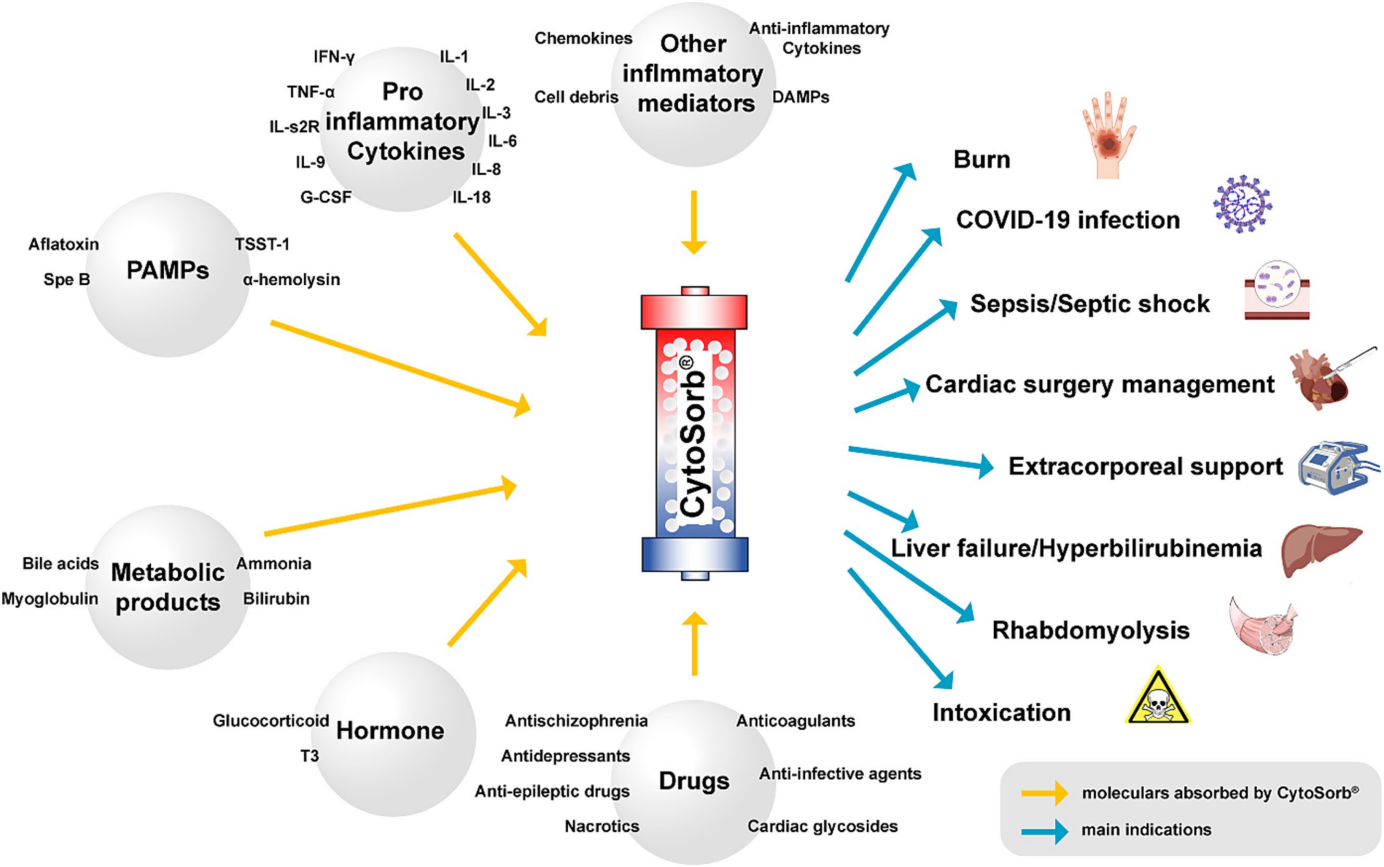
The scope of absorption and main indications for CytoSorb®. The data are summarized from in vitro studies and clinical trials. DAMPs damage-associated molecular patterns, PAMPs pathogen-associated molecular patterns, TSST-1 toxin shock syndrome toxin 1, SpeB streptococcal pyrogenic exotoxin B, T3 triiodothyronine.

Activated host immune responses can cause cytokine storms, leading to inflammatory injuries such as sepsis, post-cardiac surgery complications, and ARDS, which are the most frequent indications for CytoSorb® use (8, 126, 144). The affinity of CytoSorb® to other biomolecules, including bilirubin, bile acids, myoglobin and pharmaceutical agents, has widened its clinical application in patients with liver dysfunction, rhabdomyolysis and drug removal (Figure 4). Additionally, CytoSorb® has been also reported to treat other diseases associated with hyperinflammation, including pancreatitis (145–147) and hemophagocytic lymphohistiocytosis (148, 149). However, most of the evidence comes from case series and observational studies, with significant intergroup imbalances and baseline differences (16, 150). These limitations may be the primary reasons for current contradictory conclusions in this field. The lack of standardized RCTs further contributes to these controversies and makes it difficult to define the universal efficacy of CytoSorb® hemoadsorption in ICU settings. The uncertainty of its efficiency requires clinicians to apply this technique with caution (151, 152). Beyond these, most of small-sample observations present conflicted results with RCTs, underlying publication bias toward positive outcomes and patient selection bias may contribute to this difference. While other studies employed CytoSorb® as an adjunctive therapy for more than 3 days in peri-operative management, the REMOVE trail only utilized CytoSorb® during cardiac surgeries for a few hours (64). Beyond this, despite higher mortality in CytoSorb treatment observed in the CYCOV trial, the baseline cytokine level was not comparable (79), which assigned patients in CytoSorb treatment with sever inflammation. These findings suggested that improper on-board time, heterogeneous treatment duration and incomparable baseline condition of illness may lead to these conflicted results. Theoretically, initiating CytoSorb too late may significantly compromise its therapeutic efficacy, whereas commencing treatment too early or extending the treatment excessively may increase the risk of exacerbating infections. The variations in prescriptions based on different clinical experiences among researchers in recent studies may hinder the reliability of the final data. Consequently, reaching a standardized protocol concerning the appropriate dose, timing, equipment manufacturing and methods to patients with different indications is important. To address these issues, future studies should concentrate on a single indication under standardized study protocols, such as acutely injured individuals with hypermyoglobinemia or COVID-19 patients with a cytokine storm, to identify the most suitable syndromes or conditions for CytoSorb® treatment. Future high-quality RCTs should also consider factors such as standard study samples, change frequency of the adsorber, timing issues, medication administration and monitoring during each session and composite outcomes related to patient prognosis to better define the core aspects of CytoSorb® hemoadsorption. Academic authorities also should encourage publication of negative outcomes based on serious study design and registration of study protocols for alleviation of selective reports.

As shown in Table 3, there are several registered ongoing clinical trials^1^ aimed at evaluating the efficacy and safety of CytoSorb® in various patient populations. These include patients with septic shock (NCT04013269, NCT04963920 and NCT06261164), hyperinflammation (NCT04048434), or increased bleeding risks (NCT05077124). The trials also involve patients undergoing cardiac surgeries (NCT04596813, NCT04203004, NCT05270902 and NCT05526950) or ECMO (NCT05027529). We believe that these studies will address the current research gaps and refine clinical practice in the future.

**Table 3:** Ongoing clinical trials evaluating the safety and efficiency of CytoSorb® hemoadsorption in critical ill patients.

NCT number	Current status	Location	Study design	Study population	Key outcomes	Estimated enrollment	Dates of termination
NCT04812717	Recruiting	Netherlands	Quadruple blinded RCT	Patients with heart failure	Vascular resistance index, incidence of vasoplegia	36	31 January 2026
NCT06079021	Recruiting	Belgium	Observational study	Patients with acute on chronic live failure	serum bilirubin removal; changes in ammonia and severity of hepatic encephalopathy	20	30 June 2026
NCT05027529	Recruiting	Germany	Quadruple blinded RCT	Patients with cardiogenic shock and indication for V-A ECMO	Change in inotropic score after 72 h	54	December 2024
NCT04013269	Activate, not recruiting	Germany	Open label RCT	Patients with refractory septic shock	Percentage of patients with a reduction of catecholamine dose of at least 25% within the first 48 h of treatment; Change in organ dysfunction	32	December 2023
NCT04963920	Recruiting	Germany	Single blinded RCT	Patients treated with standard of care and vasoplegic septic shock	Percentage change in noradrenaline dose 24 h after baseline	260	May 2025
NCT04596813	Recruiting	United Kingdom	Double blinded RCT	Scheduled for elective LVAD implantation with the use of cardiopulmonary bypass	Increase in plasma IL-6 concentration and incidence of serious device related adverse events	60	30 June 2025
NCT04203004	Activate, not recruiting	Italy	Open label RCT	Patients with liver transplantation	Incidence of postreperfusion syndrome and incidence of early allograft dysfunction	20	31 December 2023
NCT05270902	Recruiting	Austria	Single blinded RCT	Adult patients undergoing heart transplantation	Difference in maximal cytokine peak levels and difference of immunosuppression	40	30 June 2024
NCT05146336	Recruiting	Germany; Italy; Portugal and Spain	Observational study	Patients who are potentially indicated to CytoSorb®	ICU mortality and in-hospital mortality	3,000	September 2032
NCT05077124	Recruiting	Austria; Belgium; Germany; Sweden and United Kingdom	Observational study	Patients with thrombotic risks	Bleeding complications including requirements for transfusions and other blood products	500	30 September 2025
NCT05526950	Recruiting	Sweden	Open label RCT	Patients undergoing double lung transplantation	Cytokine reduction and PaO2/FiO2 ratio at 24 h, 48 h and 72 h	116	31 December 2029
NCT04048434	Activate, not recruiting	Germany and Switzerland	Single blinded RCT	Patients with severe cytokine release syndrome	Levels of IL-6	34	September 2024
NCT06261164	Recruiting	Bosnia and Herzegovina	Observational study	Patients with diagnosis of SIRS, sepsis and/or septic shock and receiving treatment of amikacin and/or vancomycin	Development of population pharmacokinetic model	20	31 January 2025

RCT, randomized controlled trail, ICU, intensive care unit, SIRS, systemic inflammatory response syndrome, LVAD, left ventricle assist device, V-A ECMO, venous–arterial extracorporeal membrane oxygenation.

## 6 Conclusion

CytoSorb® has a wide range of potential indications due to its broad absorption on cytokines, bilirubin, bile acids, myoglobin and drugs. Plenty of research delivered potential application prospects of this device in rescuing critically ill patients in ICU settings. However, its efficacy and safety remain inconclusive due to the heterogeneity of current studies and a lack of high-quality randomized controlled trials. Consequently, current findings should be interpreted with caution and future investigations are necessary to address these research gaps.

## Footnotes

^1^ https://clinicaltrials.gov
